# Sensitivity of array detector measurements in determining shifts of MLC leaf positions

**DOI:** 10.1002/acm2.12148

**Published:** 2017-08-11

**Authors:** Qingyang Shang, Andrew Godley, Long Huang, Peng Qi, Ping Xia

**Affiliations:** ^1^ Department of Radiation Oncology Cleveland Clinic Cleveland OH USA

**Keywords:** Gamma index, leaf positioning shift, MatriXX, multileaf collimator (MLC), patient‐specific IMRT QA

## Abstract

Using a MatriXX 2D ionization chamber array, we evaluated the detection sensitivity of systematically introduced MLC leaf positioning shifts to test whether the conventional IMRT QA method can be used for quality assurance of an MLC tracking algorithm. Because of finite special resolution, we first tested whether the detection sensitivity was dependent of the locations of leaf shifts and positions of ionization chambers. We then introduced the same systematic leaf shifts in two clinical intensity modulated radiotherapy plans (prostate and head and neck cancer). Our results reported differences between the measured planar doses with and without MLC shifts (errors). Independent of the locations of the leaf position shifts and positions of the detectors, for the simple rectangular fields, the MatriXX was able to detect ±2 mm MLC leaf positioning shifts with Gamma index of 3%/3 mm and ±1 mm MLC leaf position shifts with Gamma index of 2%/2 mm. For the clinical plans, measuring the fields individually, leaf positioning shifts of ±2 mm were detected using Gamma index of 3%/3 mm and a passing rate of 95%. When the fields were measured compositely, the Gamma index exhibited less sensitivity for the detection of leaf positioning shifts than when the fields were measured individually. In conclusion, if more than 2 mm MLC leaf shifts were required, the commercial detector array (MatriXX) is able to detect such MLC positioning shifts, otherwise a more sensitive quality assurance method should be used.

## 1 INTRODUCTION

With the application of stereotactic body radiotherapy (SBRT) to prostate cancer, further reduction of inter‐ and intrafraction prostate motion becomes important.^1^, ^2^, ^3^, ^4^ Even with daily diagnostic image quality‐guided soft tissue alignment, our previous study found that 6/4 mm (4 mm posterior) planning margin is necessary to account for residual interfraction errors, such as prostate rotation and deformation.^5^ To minimize intrafraction prostate motion, daily insertion of an endorectal balloon has been clinically adopted, but the insertion of the rectal balloon may introduce a larger interfractional prostate motion. Jones et al. reported that 69% of fractions required insertion adjustments of the endorectal balloon in order to reduce prostate rotation and deformation.^6^ Without endorectal balloon, the intrafraction prostate motion is sporadic, depending on the treatment duration,^7^, ^8^ and thus compensating the intra‐fraction prostate motion is treatment modality‐dependent. Based on the data collected from the Calypso system, if the treatment duration is less than 4 min, 2 mm planning margins in the longitudinal and vertical directions were proposed with the assumption of a minimal lateral intrafraction motion.^7^

To further reduce the planning margin to account for interfraction prostate deformation or rotation larger than 3° (a typical range a robotic table can compensate for), some researchers proposed real‐time adaptive planning,^9^ which may encounter logistical and practical challenges.^10^ Others proposed to shift MLC leaves to track changes of the prostate. To account for the independent prostate and pelvic lymph nodal movement, our group also proposed an MLC tracking method to compensate for interfraction prostate motion.^11^ To account for intrafraction prostate motion, a real‐time monitoring with frequent patient repositioning or with MLC tracking during treatment has been proposed. A prototype MLC tracking system has been developed and clinically tested by Keall group.^2^, ^4^ To experimentally validate the MLC tracking algorithm for interfraction or intrafraction motion, we designed this study to test whether a commonly used detector array device is sufficient to verify the MLC tracking algorithm.

## 2 MATERIALS AND METHODS

### 2.A MLC tracking algorithm

An MLC tracking algorithm was proposed by our group to compensate for independent movement of the prostate and pelvic lymph nodes (PLN). With this MLC tracking method, the displacement of the prostate was compensated without affecting the dose distributions to PLN. Briefly, the algorithm was implemented with an in‐house program that automatically identifies MLC leaf pairs that were collimated to the prostate in the planning CT and adjusts the positions of these leaf pairs for each segment of the IMRT plan to compensate for the interfraction prostate motion relative to the pelvic bones. Meanwhile, the MLC leaves that were conformal to the PLN were unchanged. Based on the magnitude and direction of the daily prostate movement, the algorithm adjusts the positions of selected MLC leaf pairs to follow the translational motion of the prostate for each beam. The algorithm assumes that the prostate is a rigid body and the rotational motion is negligible. Because the field size in unchanged and the changes in the off‐axis factors contribute only a negligible amount to the dose distribution, this MLC tracking does not require a real‐time dose calculation.

### 2.B Testing fields

A single rectangular field (2.4 × 10.4 cm^2^), one 5‐field prostate IMRT plan, and one 9‐field head and neck IMRT plan, were used. The single rectangular field represents a “best case” for the Gamma index to detect the leaf positioning shifts, as opposed to the more complicated IMRT plans. It is also used to assess the effect of the MatriXX array resolution. The IMRT plans were created using direct machine parameter optimization (DMPO) with beam energy of 6 MV within the Pinnacle treatment planning system (Pinnacle 9.0, Philips Radiation Oncology System, Madison, WI, USA). The prescribed fractional dose for the two IMRT plans was 2 Gy to the clinical target volume (CTV).

### 2.C MLC leaf positioning shifts

Within Pinnacle, the positions of the MLC leaves on one side of the leaf bank in each segment were shifted by ±1, ±2, ±3, or ±4 mm, while keeping the rest of the treatment plan parameters unchanged. Nine plans were created for each patient: the original plan without leaf positioning shifts, and eight plans with increasing systematic shifts. All these plans were measured and compared.

### 2.D Measurements

All dose measurements were conducted with the MatriXX 2D array, which consists of 1020 air‐vented parallel plate ionization chambers on a 32 × 32 Cartesian grid (there are no ion chambers at the four corners of the array). The physical and dosimetric properties of this device have been reported previously.^12^, ^13^, ^14^, ^15^, ^16^, ^17^ The chamber center‐to‐center separation is 0.762 cm, with a sensitive volume of 0.08 cm^3^ (0.45 cm in diameter and 0.5 cm in height), covering a 24.4 × 24.4 cm^2^ active area. The MatriXX device was positioned at the isocenter of the linear accelerator using the room lasers.

All plans were delivered in step‐and‐shoot mode on an Elekta linear accelerator (Elekta, UK) equipped with 40 pairs of MLC leaves, with a projected leaf width of 4 mm at the isocenter. MLC calibration was performed before the measurements and confirmed by a picket fence test. For the rectangular field, dose distributions were measured at 0° gantry angle. For the IMRT plans, dose distributions were measured at both the treatment gantry angles and at 0° fixed gantry angle. In addition, the planar doses from individual and composite fields for the IMRT plans were measured.

### 2.E Effect of the ion chamber resolution of the MatriXX

We assessed the sensitivity of MatriXX to leaf positioning shifts with respect to the position of the shifts relative to the detector columns. For a centered 2.4 × 10.4 cm^2^ field, we measured dose distributions with the MatriXX aligned with the machine isocenter at coordinate (0, 0) cm. The center of the rectangular field was then shifted to (−0.8, 0.0) cm in 1 mm intervals along the cross plane. The right edge of the field on the horizontal central axis moved correspondingly from the initial position of (1.2, 0.0) cm to (0.4, 0.0) cm. For all fields, a 1 mm leaf positioning shift was present at the right field edge (shifting the MLC leaves on the right leaf bank inward by 1 mm). Thus, the 1 mm field edge shift was measured at varied locations between the two adjacent ion chamber columns at 1.143 cm and 0.381 cm away from the in‐plane center of the MatriXX. This is also the reason we chose 2.4 cm as the field width (so 1.2 cm half width includes two ion chamber columns). We chose 26 pairs of MLC symmetrically with 4 mm leaf width, so the field height was 10.4 cm. Gamma index was computed between the measured planar doses with and without shifts for each field.

### 2.F Gamma index computation

Gamma index was computed using the OmniPro I'mRT analysis software. When assessing the effect of MatriXX array resolution using the single rectangular field, Gamma index was directly computed between the measured planar doses with and without leaf positioning shifts to eliminate other measurement uncertainties (such as MatriXX setup shifts and detector volume averaging effect) and potential beam modeling errors from the TPS. For clinical IMRT plans with multiple fields, Gamma index was computed between the measured planar dose with introduced leaf positioning shifts and the calculated planar dose from TPS free of shift, as in routine patient‐specific IMRT QA. A sudden drop of Gamma passing rate from the baseline value (where no leaf positioning shift was introduced) indicates potential MLC leaf positioning shifts. In this study, we chose 95% as the threshold of Gamma passing rate for shift identification.

A threshold of 10% of the maximum dose was used to exclude areas with low dose from Gamma index calculation. Planar dose distributions from measurements were interpolated to a resolution of 1 by 1 mm^2^ within the OmniPro I'mRT analysis software to match those from the TPS.

## 3 RESULTS

### 3.A A simple rectangular field

**Figure 1:**
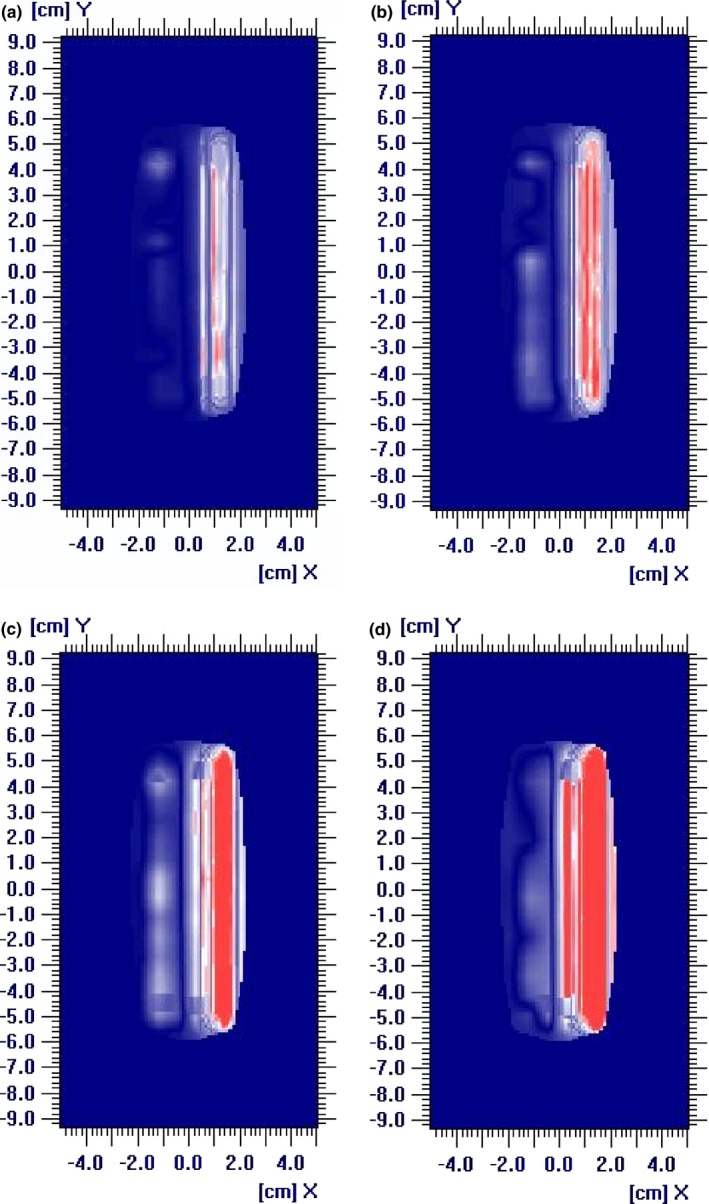
Gamma indices of 3%/3 mm of the single rectangular field with MLC leaf positioning shifts up to −4 mm. (a) shift = −1 mm, γ = 97.5%; (b) shift = −2 mm, γ = 84.2%; (c) shift = −3 mm, γ = 78.3%; (d) shift = −4 mm, γ = 66.3%.

Table 1 shows Gamma indices of 3%/3 mm and 2%/2 mm computed between the measured planar doses with and without MLC leaf positioning shifts of up to ±4 mm on one side of the leaf bank for the 2.4 × 10.4 cm^2^ single rectangular field at 0° gantry angle. Figures 1 shows Gamma indices of 3%/3 mm with leaf positioning shifts from −1 mm to −4 mm. With a threshold of a passing rate of 95%, MatriXX can detect ±2 mm shifts with Gamma index of 3%/3 mm and ±1 mm shifts with Gamma index of 2%/2 mm.

**Table 1:** Gamma indices of 3%/3 mm and 2%/2 mm computed between the measured planar doses with and without MLC leaf positioning shifts up to 4 mm for a 2.4 × 10.4 cm^2^ single rectangular field at 0° gantry angle

Gamma index criterion (%)	Shift (mm)								
	−4	−3	−2	−1	0	+1	+2	+3	+4
3%/3 mm	66.3	78.3	84.2	97.5	100	95.2	87.2	74.1	66.3
2%/2 mm	53.9	60.5	72.1	82.4	100	85.1	69.8	60.8	58.1

### 3.B Effect of ion chamber resolution and position

The 2%/2 mm Gamma index passing rate was observed to be consistent at (82.7 ± 1.8) % regardless of the relative position of the −1 mm leaf positioning shift to the detectors.

### 3.C Clinical IMRT treatment plans

#### 3.C.1 IMRT prostate plan measured at the treatment gantry angle

**Figure 2:**
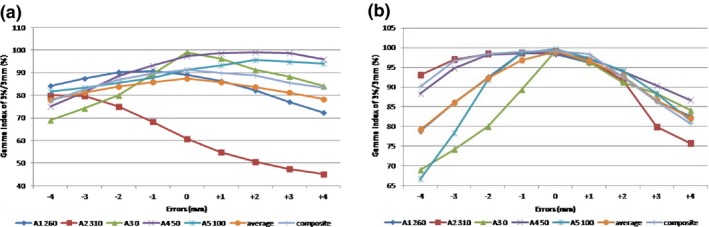
The change of Gamma index of 3%/3 mm with different amount of MLC leaf positioning shifts for each individual and composite field of a clinical prostate IMRT plan. Gamma index was computed from comparison between the measured planar doses with shifts and the calculated doses without shifts at (a) the treatment gantry angles, and (b) 0° gantry angle.

The Gamma indices of individual fields, their average and the indices of the composite plan delivered at the treatment gantry angles of the prostate plan are shown in Fig. 2(a). Gamma index was computed from comparisons between the measured planar doses with leaf positioning shifts and the calculated doses without shifts. Because of the angular dependence of the MatriXX device, the Gamma index for some fields (such as A2, A4, and A5) increased as the MLC leaf positioning shifts increased. Without leaf positioning shift, the average Gamma indices of individual fields measured at the delivered gantry angles were only (87.5 ± 15.5)% and (63.8 ± 18.1)% at 3%/3 mm and 2%/2 mm, respectively. Gamma indices of the composite field of 3%/3 mm and 2%/2 mm were 91.3% and 71.7%, respectively.

#### 3.C.2 IMRT prostate plan at 0° fixed gantry angle

**Figure 3:**
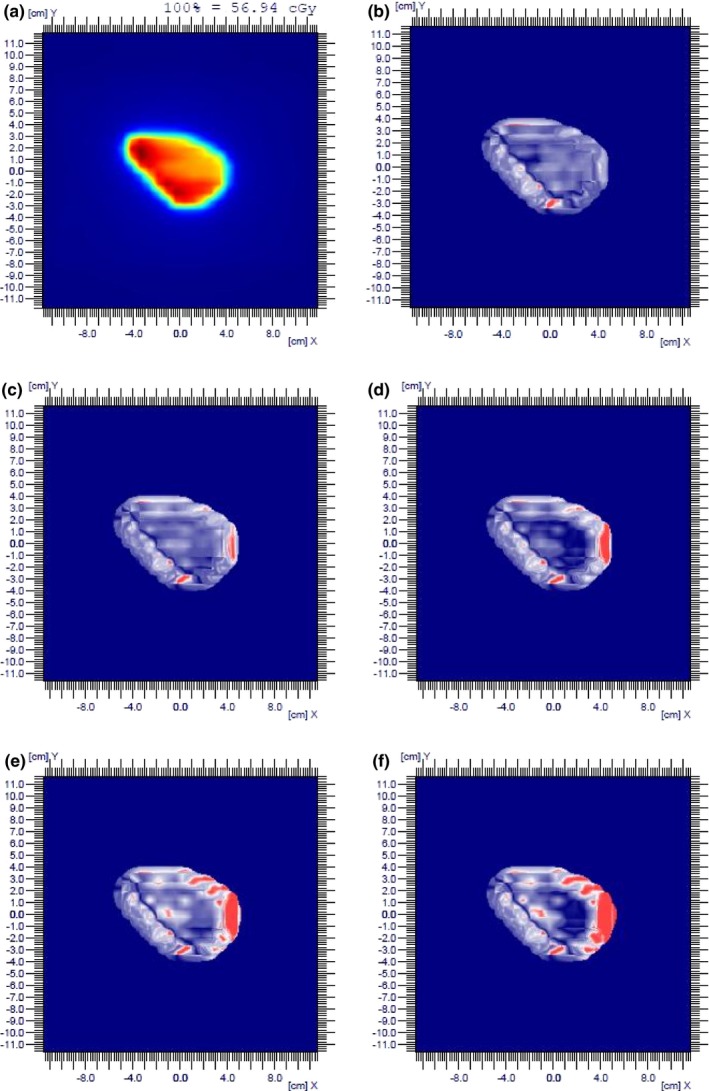
An example of a prostate IMRT field at 0° beam angle and Gamma indices of 3%/3 mm when leaf positioning shifts up to 4 mm were introduced. (a) planar dose of the field; (b) shift = 0 mm, γ = 98.4%; (c) shift = 1 mm, γ = 96.1%; (d) shift = 2 mm, γ = 92.7%; (e) shift = 3 mm, γ = 86.5%; (f) shift = 4 mm, γ = 82.5%.

To eliminate the angular dependence of the MatriXX array, dose distributions were measured at 0° fixed gantry angle. Table 2 and Fig. 2(b) show the Gamma indices of individual fields, their average and Gamma indices of the composite fields with all beams delivered at 0° gantry angle. With ±2 mm shift introduced, the average Gamma indices of 3%/3 mm were 92.8% and 92.2%, respectively, 6.1% and 6.7% decrease from the baseline 98.9%; and the average Gamma indices of 2%/2 mm were 72.1% and 70.4%, respectively, 10.5% and 12.2% decrease from the baseline 82.6%. Figure 3 depicts an example of Gamma indices of 3%/3 mm of a prostate field at beam angle 0° when MLC leaf positioning shifts up to 4 mm were introduced. Table 2 shows that detecting a 2 mm MLC leaf positioning shift in prostate patient‐specific IMRT QA with MatriXX requires a Gamma index of 3%/3 mm and a tight passing rate of 95% for individual fields.

**Table 2:** The average and standard deviation (SD) of Gamma indices of individual fields and Gamma indices of the composite field of 3%/3 mm and 2%/2 mm computed between the measured planar doses with MLC leaf positioning shifts up to 4 mm and the calculated doses without shifts of the prostate IMRT plan with all beams at 0° gantry angle

Gamma index criterion (%)	Shift (mm)								
	−4	−3	−2	−1	0	+1	+2	+3	+4
Average of individual fields									
3%/3 mm	79.2	86.1	92.2	96.8	98.9	96.6	92.8	86.7	82.1
3%/3 mm (SD)	11.6	9.9	7.5	4.2	0.5	0.5	1.3	4.0	4.1
2%/2 mm	55.3	61.9	70.4	78.3	82.6	77.9	72.1	64.5	61.5
2%/2 mm (SD)	8.7	10.2	8.5	7.9	5.3	4.9	5.6	8.9	7.2
Composite field									
3%/3 mm	90.1	96.7	98.3	99.0	99.2	98.4	92.6	86.4	80.7
2%/2 mm	62.3	69.8	80.5	84.6	85.4	76.3	68.7	62.6	55.8

Table 2 and Fig. 2(b) also shows that the Gamma index of the composite field was generally greater than the average Gamma index of individual fields and thus less sensitive to leaf positioning shifts. Figure 2(b) further shows that for this particular plan, the average Gamma index of 3%/3 mm exhibited comparable sensitivity to both positive and negative leaf positioning shifts, while composite field Gamma index was more sensitive to positive shifts. This further implies the poor suitability of composite field measurement to leaf shift detection.

#### 3.C.3 IMRT head and neck plan at 0° fixed gantry angle

**Figure 4:**
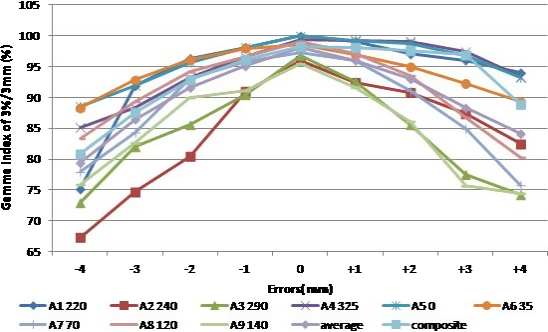
The change of Gamma index of 3%/3 mm with different amount of MLC leaf positioning shifts for each individual and composite field of a clinical head and neck IMRT plan. Gamma index was computed from comparison between the measured planar doses with shifts and the calculated doses without shifts at 0° gantry angle.

**Figure 5:**
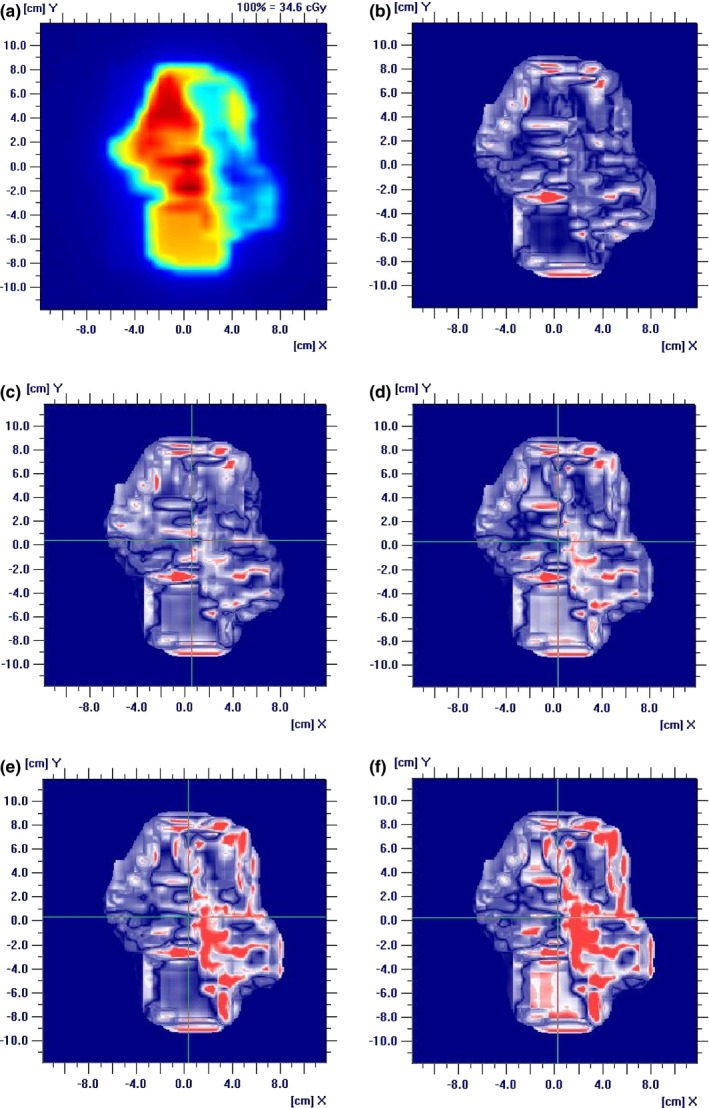
An example of a head and neck IMRT field at 0° beam angle and Gamma indices of 3%/3 mm when leaf positioning shifts up to 4 mm were introduced. (a) planar dose of the field; (b) shift = 0 mm, γ = 97.3%; (c) shift = 1 mm, γ = 95.8%; (d) shift = 2 mm, γ = 93.0%; (e) shift = 3 mm, γ = 84.3%; (f) shift = 4 mm, γ = 77.9%.

The average Gamma indices of individual and composite fields and Gamma with all beams at 0° gantry angle for the head and neck IMRT plan are shown in Table 3 and Fig. 4. When ±2 mm shifts were introduced, the average Gamma index of 3%/3 mm decreased from the baseline 98.1% to 93.0% and 91.6% (5.1% and 6.5% decrease); and the average Gamma index of 2%/2 mm decreased from the baseline 83.4% to 76.4% and 72.7% (7% and 10.7% decrease). Figure 5 shows Gamma indices of 3%/3 mm of a head and neck field at beam angle 0° when MLC leaf positioning shifts up to 4 mm were introduced. Similar to the prostate plan, 2 mm MLC leaf positioning shifts can be identified by patient‐specific IMRT QA for the head and neck plan with MatriXX device when using a Gamma index of 3%/3 mm and a 95% passing rate. The composite field Gamma index of the head and neck plan showed a similar asymmetric property as the prostate plan.

**Table 3:** The average and standard deviation (SD) Gamma indices of individual fields and Gamma indices of the composite field of 3%/3 mm and 2%/2 mm computed between the measured planar doses with MLC leaf positioning shifts up to 4 mm and the calculated doses without shifts of the head and neck IMRT plan with all beams at 0° gantry angle

Gamma index criterion (%)	Shift (mm)								
	−4	−3	−2	−1	0	+1	+2	+3	+4
Average of individual fields									
3%/3 mm	79.4	86.5	91.6	95.1	98.1	96.0	93.0	88.3	84.1
3%/3 mm (SD)	7.3	6.0	5.4	3.3	1.7	3.1	5.0	8.1	8.4
2%/2 mm	57.5	64.6	72.7	78.6	83.4	81.4	76.4	68.8	65.6
2%/2 mm (SD)	7.8	6.12	7.1	7.6	6.2	8.7	12.0	13.1	10.5
Composite field									
3%/3 mm	80.8	87.6	92.9	96.1	98.3	98.2	97.6	96.9	88.9
2%/2 mm	64.0	72.3	79.6	87.4	93.0	92.2	91.1	79.8	68.6

## 4 DISCUSSION AND CONCLUSION

In this study, sensitivity of detecting MLC leaf positioning shifts was assessed using the MatriXX ionization chamber array, which is a conventional IMRT QA method used in our practice. Our study has shown that with a threshold of 95% Gamma passing rate, MatriXX 2D array can detect 2 mm leaf positioning shift with 3%/3 mm criterion and 1 mm shift with 2%/2 mm criterion for a simple rectangular field. For both prostate and head and neck IMRT plans, measurement of individual field measured at 0° gantry angle can detect 2 mm shift with MatriXX using 3%/3 mm Gamma index with a passing rate of 95%. Gamma index of composite fields at the delivery gantry exhibited much less sensitivity to the MLC shifts. If an adaptive plan adjusted MLC leave positions smaller than 2 mm, the MatriXX experimental verification method is not sensitive enough to detect the changes. Compared to other measurement devices such as film, MapCheck, ArcCheck, and EPID, the MatriXX detection sensitivity of MLC displacements of 2 mm is similar to the results of these methods (see more discussion below). Alternative methods to validate MLC shifts smaller than 2 mm could be the log file analysis method. In this study, we introduced leaf positional displacements through uniformly shifting only one bank of MLC leaves. With MLC tracking algorithm, the MLC shifts would occur to the both banks of MLC leaves. Our choice of shifting one bank of MLC leaves uniformly was to simplify our analysis. With MLC leaves shifted 1 mm in both banks, the Gamma passing rate would be similar to 2 mm shifts from one leaf bank. Because of prostate rotation and deformation, MLC leaves may not be shifted uniformly. Uniform shifts for all involved MLC leaves in the IMRT plans assume the treatment targets be rigid, which also simplified our analysis.

While our initial purpose of this study was to experimentally validate our MLC tracking algorithm for prostate plans, we expanded our experiment to a more complex HN IMRT plan to investigate whether including more number of segments with larger segment areas in HN plans would change the detection sensitivity. Our measurement results did not show correlation of between the plan complexity and detection sensitivity, albeit only one plan for prostate and one plan for HN were tested. Further research is required. For small field SBRT cases, such as lung, applying MLC tracking could be challenging due to much fast tumor motion cycle when compared to the prostate motion. Because of small number of leaves involved in each segment, we anticipate the detection sensitivity for MLC leaf displacements would be the same or worse.

The detection sensitivity of MLC leaf positioning shifts has been studied with different devices.^18^, ^19^, ^20^, ^21^, ^22^ Yan et al.^18^ found that film and the MapCHECK diode array could detect systematic leaf positioning shifts on the order of 2 mm with both absolute distance‐to‐agreement (DTA) and Gamma index analysis using both 2%/2 mm and 3%/3 mm criteria based on a field‐by‐field planar dose comparison with all beams at 0° gantry angle for head and neck treatments. Garcia‐Vicente et al.^19^ showed systematic outward MLC gap width shifts up to 2 mm could not be detected with ArcCHECK diode array using Gamma criterion 3%/2 mm for true composite dose analysis of both prostate and head and neck treatment, whereas an absolute dose measurement with the criterion of 2% at isocenter using an inserted micro‐ionization chamber was able to identify 1 mm MLC gap shifts. Rangel et al.^20^ reported the criteria most sensitive to detect the MLC leaf shifts were 3% absolute dose difference, 3 mm DTA for MapCHECK, the Gamma index with 2%/2 mm for the EPID. Heilemann et al.^21^ conducted a sensitivity study with both ionization chamber and diode 2D array devices for prostate and head and neck VMAT delivery, and suggested Gamma index of 2%/2 mm with passing rate greater than 90% was necessary to detect leaf positioning shifts of 1 mm on both MLC banks. Unlike other studies mentioned above, which were based on planar doses comparison between measurement and calculation, Tatsumi et al.^22^ proposed a direct method for evaluating the impact of the leaf positioning shifts on dose distribution for prostate VMAT delivery by comparing the measured planar doses with and without shifts. They concluded that the MLC shift influence on dose distribution depended on the optimization results of the treatment planning system (TPS), in particular the average leaf gap width. The tolerance limits of the leaf positioning shifts to maintain the passing rate under a dose difference criterion of 2% for three treatment planning systems, Monaco (Elekta, USA), SmartArc (Philips, USA), and Ergo (Elekta, Italy), were 0.3, 0.5, and 1.0 mm, respectively.

The MatriXX has a known issue called volume averaging effect. Due to the finite size of the ion chambers, the secondary electrons produced in materials between the chambers can travel into the air‐filled volumes of the chambers, exerting non‐negligible signal perturbation in dose measurement. Because of this volume averaging effect, the point dose from the measurement deviates from the point dose from TPS calculation, especially in high dose regions. Some studies have shown that after convolution of the calculated dose with the lateral response of the ion chamber, the corrected planned dose distribution had better agreement with the measured dose distribution, resulting in a higher Gamma passing rate.^14^, ^15^ Direct comparison between the measured planar doses with and without shifts for Gamma index can bypass this convolution correction and may provide an effective way to assess the consequence of detector resolution on sensitivity of MatriXX to leaf positioning shifts. This also excludes errors from beam modeling in the TPS. So when determining the sensitivity of MatriXX to leaf positioning shifts with respect to their relative positions to detectors, measurements with and without shifts were compared directly. Gamma index evaluated in patient specific IMRT QA is however usually obtained from dose comparison between measurement and calculation, so the measured planar doses with leaf positioning shifts were compared with the calculated planar doses without shifts for clinical IMRT plans.

It has been reported that MatriXX has an inherent angular dependence of up to 11%.^16^ The angular dependence causes dose shifts when MatriXX is positioned on the treatment couch and the gantry is angled. When Gamma index is obtained from planar dose comparison between measurement and calculation at treatment angles, the inconsistency between the variation of Gamma index and the increment of shift magnitude shown in Fig. 2(a) is the result of the complex interplay between dose shifts caused by MatriXX angular dependence and MLC leaf positioning shifts. Setting the gantry angle to 0° for each field removes the angular dependence, thus the variation of Gamma index solely reflects the influence of the introduced leaf positioning shifts. Our study also shown that Gamma index of the composite field at fixed gantry angle exhibited much less sensitivity to the leaf positioning shifts. This is because errors from individual fields could compensate each other in the composite field. For a particular IMRT plan, the compensation effect might be considerable for errors of one sign but moderate or weak for errors of the other sign.

The effect of dose grid resolution for dose calculation in the Pinnacle TPS on Gamma index was evaluated for the prostate composite fixed gantry field. Planar doses calculated with both 3 × 3 mm^2^ and 2 × 2 mm^2^ dose resolutions in the TPS were compared with the measured planar doses for Gamma index computation. Results show that little improvement could be achieved from increasing the dose grid resolution.

A threshold of 10% of the maximum dose within the field was used to limit the points considered in the Gamma analysis. More specifically, Gamma passing rate represents the percentage of points that satisfies a certain criterion over the area in which the point dose is greater than 10% of the maximum dose, instead of over the whole MatriXX area. This reduces the favorable bias for the inclusion of large amount of low dose points. As a result, the changing trend of Gamma index as the shift magnitude increases was more noticeable.

Some studies have shown that many of the clinically critical shifts may not be detected using commonly accepted Gamma metrics. Nelms et al.^23^ simulated four types of beam modeling shifts including MLC transmission factor shifts and beam penumbra shifts. Comparing between the shift‐induced plans with the shift‐free plans, they found only weak correlations between the conventional IMRT QA performance metric of Gamma index and the anatomy‐based clinically relevant dose difference metrics (such as CTV D95, mean dose, etc.). Kruse et al.^24^ investigated the sensitivity of Gamma metrics of 3%/3 mm and 2%/2 mm in differentiating clinically acceptable and unacceptable plans using an EPID and MatriXX ion chamber array. It was observed that Gamma analysis of single field measurements failed to identify important dosimetric inaccuracies of the overall plan. Our study focused on MLC leaf positioning shifts being present in treatment delivery. In conclusion, with a passing rate of 95%, using a commercial detector array (MatriXX) can detect 2 mm MLC leaf positioning shifts using 3%/3 mm Gamma criterion, measured on individual fields at 0° gantry angle. If smaller than 2 mm MLC leaf shifts were required, a more sensitive quality assurance method should be used.

## CONFLICT OF INTEREST

The authors declare no conflict of interest.
